# Homogeneity effects in natural language semantics

**DOI:** 10.1111/lnc3.12350

**Published:** 2019-11-10

**Authors:** Manuel Križ

**Affiliations:** ^1^ Department of Linguistics University of Vienna Vienna, Austria

## Abstract

Natural language sentences in which a property is ascribed to a plurality of objects have truth conditions that are not complementary with the truth conditions of the negations of such sentences. Starting from this observation, this paper presents an overview of so‐called *homogeneity effects*. Arguably a pervasive feature of natural language, homogeneity has reflexes in various domains and opens up a prospect for a unified analysis of phenomena that were hitherto viewed in quite different terms.

## 1 INTRODUCTION

It is an observation going back to Fodor, 1970 that sentences with definite plurals and their negations do not have complementary truth conditions, i.e. there are situations where neither sentence is true:

(1) a. Mr. Benfleet published the books.
  ⇝ He published (pretty much) **all** of them.
  b. Mr. Benfleet didn't publish the books.
  ⇝ He published (pretty much) **none** of them.

This effect has come to be known as *homogeneity*, because the affirmative (1a) is true if Benfleet published all the books, while its negation (1b) is true if he published none; in both cases, the group of books is homogeneous with respect to the property of having been published by Benfleet.

In contrast, sentences with *all*‐phrases instead of plain definite plurals do not show homogeneity: the negated sentence is true whenever the affirmative sentence is not true.

(2) a. Mr. Benfleet published all the books.
  b. Mr. Benfleet didn't publish all the books.

The purpose of this article is to give an overview of this phenomenon and its various reflexes in sentences with plural noun phrases but also beyond. Throughout, we try to remain as descriptive as possible, except where such strong arguments are available that a theoretical question can be regarded as settled. However, in some instances, the ways in which competing theories conceive of the phenomena are so different that it is difficult to abstract over all of them; in this case, we allow ourselves a more theory‐laden mode of presentation. Note that we have to presuppose a basic familiarity with how plural predication is standardly handled in linguistic semantics following Link, 1983.

## 2 HOMOGENEITY AS A KIND OF GAPPINESS

Take it as a matter of definition for the purposes of this article that we call a sentence *false* whenever its negation is true. We can thus say that the sentences (1) are neither true nor false in a situation where Benfleet published only some of the books. From hereon, we will say that a sentence is *undefined* when it is neither true nor false. A sentence that is sometimes undefined will be called *gappy* because there is, as it were, a gap between the region of logical space in which it is true and the region where it is false. Note that we mean for these terms to be descriptive and to be understood in as theory‐neutral a way as possible. 1In some, but not all theories of homogeneity, these terms do have an independent theoretical meaning. Most explicitly, Križ (2015a) posits truth, falsity, and undefinedness as theoretical primitives and analyses homogeneity with the tools of trivalent logic.


### 2.1 Homogeneity violations

When a sentence is undefined in the way that homogeneity brings about, we also speak of a *homogeneity violation* (because the plurality in question is not homogeneous with respect to the property ascribed to it). Undefinedness due to a homogeneity violation has a certain particular signature.

One finds experimentally that homogeneity violations are associated with a variability in judgements that sets them apart from both true and false sentences. Schwarz (2013) found that in a truth‐value judgement task with the two answer options *true* and *false*, undefined affirmative sentences received varying responses. Negated sentences did not feature in the experiment. Križ and Chemla (2015) presented both affirmative and negated sentences with the three answer options *completely true*, *completely false*, and *neither*. For undefined sentences, answers were predominantly split between *completely false* and *neither*, with only a small proportion of *completely true* responses, whereas sentences that were either true or false received very consistent responses.

Furthermore, while undefinedness is not always introspectively accessible, it sometimes gives rise to a feeling of hesitation, and it is reflected in what sort of response it is natural to give to a sentence. The most natural way to react to an undefined sentence is generally *well* followed by a correction, whereas both *yes* and *no* may under certain circumstances be possible but tend to be dispreferred. 2Note that *well* also has other uses and is not specific to homogeneity violations. Furthermore, not all languages appear to have an item that functions like English *well* as a reaction to homogeneity violations.

(3) Context: *Mr. Benfleet published half of the books in question.*
  A: Mr. Benfleet published the books.
  B: Well, half of them.
  B′: ??Yes, half of them.
  B″: ??No, only half of them.

In addition, the *well* can be drawn‐out and pensive as if the respondent was trying to decide how much credit to give the speaker for their neither true nor false utterance.

(4) A: Mr. Benfleet published the books.
  B: Weeell…Half of them, anyway.

### 2.2 Varieties of gappiness

There are, however, many other types of sentences in natural language that are gappy in this purely descriptive sense, including sentences with presuppositions, those with implicatures, free‐choice sentences, and sentences with vague predicates.

(5) **Presuppositions**
  a. Mary stopped smoking. ⇝ Mary used to smoke.
  b. Mary didn't stop smoking. ⇝ Mary used to smoke.
**neither** if Mary never smoked
(6) **Quantity implicatures**
  a. Mary saw John or Bill. ⇝ She saw one but not both.
  b. Mary didn't see John or Bill. ⇝ She saw neither of the two.
**neither** if Mary saw both John and Bill
(7) **Free‐choice sentences**
  a. Mary may eat an apple or a pear. ⇝ She can choose either.
  b. Mary may not eat an apple or a pear. ⇝ She can choose neither.
**neither** if she can eat an apple, but not a pear (or the other way around)
(8) **Vagueness**
  a. John is tall.
  b. John is not tall.
**neither** if he is only sort of tall (i.e., a borderline case)

It is currently an open question which, if any, of these phenomena are to be unified on the theoretical level. While initial discussions of homogeneity (Gajewski, 2005; Löbner, 2000; Schwarzschild, 1996) assumed, largely for lack of a better alternative, that it was presuppositional, this notion has been abandoned as marked differences between presuppositional and homogeneous sentences were pointed out (Križ, 2015a; Spector, 2013).

Consider first the issue of projection. Presuppositions project universally from the scope of universal quantifiers, and they are left untouched by negation. For example, both (9a) and its negation (9b) presuppose that all of John's children used to smoke.

(9) a. All of John's ten children stopped smoking.
  b. Not all of John's ten children stopped smoking.
  ⇝ All of John's ten children used to smoke.

If *read the books* had a homogeneity presupposition, then this presupposition should project analogously, and both (10a) and (10b) should entail that none of John's children read only some of the books.

(10) a. All of John's ten children read the books.
  b. Not all of John's ten children read the books.

This is correct for the affirmative (10a) but not for its negation (10b). (10b) is true as soon as one of John's children read none of the books and does not carry an additional entailment that none of the children read only some of the books (see Križ and Chemla, 2015 for experimental data confirming this).

The ways in which it is possible and natural to react to a presupposition failure are also starkly different from those for homogeneity violations. Presupposition failures can be objected to with *hey, wait a minute*, as in (11). This is also possible, if not very cooperative, for presuppositions that one does not know to be false, but which were not previously established as true, as in (12).

(11) A: Does John know that Mary bought either all the jewels or none of them?
  B: Wait a minute! She actually bought only some of them.
(12) A: Does John know that Mary bought either all the jewels or none of them?
  B: Wait a minute! I didn't know she couldn't possibly have bought only some of the jewels.

Spector (2013) points out that the putative homogeneity presupposition does not behave like this:

(13) A: Did Mary buy the jewels? B: #Wait a minute! I didn't know she couldn't possibly have bought only some of the jewels.

Furthermore, the pensive *weeell* response is quite odd with a presupposition failure (Križ, 2015a): 3This is to be distinguished from a very short *well*, which apparently has a different function and is acceptable with presupposition failures:(i) A: Mary stopped smoking. B: Well, she never smoked in the first place.

(14) A: Mary stopped smoking.
  B: ?#Weeell…She never smoked in the first place, actually.

Experimental results also indicate a difference between homogeneity violations and presupposition failures (Cremers et al., 2017; Zehr, 2014).

Newer analyses that build on Schwarzschild and Löbner instead treat homogeneity as something *sui generis* while noting parallels with vagueness (Križ, 2015a; Križ & Spector, 2017; see Cremers et al., 2017 for relevant experimental evidence). 4Krifka, 1996, an additional precursor of Križ and Spector, 2017, attempts to treat definite plurals as subject to a kind of ambiguity but does not situate this in the wider landscape of phenomena.Meanwhile, Magri (2014) attempts to assimilate homogeneity to implicatures in a particular way but see Križ 2015a for counterarguments. Recently, Bar‐Lev (2018) has likened homogeneity effects to free‐choice effects, both of which are in turn viewed as a special kind of implicature. A proper discussion of the relevant considerations is beyond the scope of this paper, in which we will therefore proceed to regard homogeneity as a phenomenon of its own.

## 3 HOMOGENEITY AS A PROPERTY OF PREDICATES

It was initially assumed that homogeneity was a property of distributive predication, and some authors have explicitly posited that it is the silent distributivity operator that introduces this particular kind of gappiness (Gajewski, 2005). However, Križ (2015a) points out that homogeneity can also be observed with collective predicates. (15a) conveys that there is a performance of the play *Hamlet* in which all and only the boys are participating. Its negation (15b), however, isn't rendered fully true if just a few boys are doing something else, or if there are a few girls who are also participating. Rather, it requires something much stronger that none of the boys are participating in a performance of *Hamlet*.

(15) a. The boys are performing *Hamlet*. ⇝ The play is being performed by all and only the boys.
  b. The boys aren't performing *Hamlet*. ⇝ None of the boys are participating in a performance of the play.

We thus need a way to state homogeneity that applies both to distributive and to collective predication. Križ (2016) offers a generalisation that, simplified slightly, amounts to the following:

(16) **Homogeneity generalisation** (adapted from Križ, 2016)
  A homogeneous predicate that is not true of a plurality *a* is undefined of *a* if it is true of some plurality *b* that overlaps (i.e., has parts in common) with *a*.

It is useful to decompose this into three cases:

(17) a. *b* is a proper part of (i.e., fully contained in) *a* (*downwards homogeneity*)
  b. *b* is a proper superplurality of (i.e., fully contains it) *a* (*upwards homogeneity*)
  c. *a* and *b* have parts in common, but neither contains the other (*sideways homogeneity*)

For distributive predicates, which are true of a plurality if and only if they are true of all of its parts, these three types of configurations coincide, but they are distinguishable with collective predicates. Note that we are operating on a purely descriptive level here; existing theories differ in whether upwards and downwards homogeneity are fundamentally separate, but on a par (Križ, 2015a), or one is ultimately reducible to the other (in particular, Bar‐Lev 2018 attempts to reduce upwards homogeneity to downwards homogeneity).

Consider again the sentence in (18). This sentence is true if there is a performance of *Hamlet* going on in which all and only the boys participate, and we predict it to be false only if no boy is taking part in any performance of the play. There are several types of scenarios in which neither one nor the other is the case and the predication is forced to be undefined by homogeneity.

(18) The boys are performing *Hamlet*.

Scenario 1 *Only a subgroup of the boys is staging the performance.*

(18) isn't true in such a scenario, 5Putting aside the phenomenon of *team credit*, which Križ (2016) argues is an instance of non‐maximality, cf. Section 7 below.but it certainly isn't false, either. Being true not of the plurality of all boys, but of a plurality that overlaps with them (in this case, a proper part), the predicate is constrained to be undefined due to downward homogeneity.

Scenario 2 *All students together, including the boys, are performing the play.*

Here, the boys are performing *in Hamlet*, but we would not call (18) true. 6Some collective predicates, like *carry a piano* and even more easily *play the Kreutzer sonata*, seem to be able to undergo reinterpretation to yield a participatory reading, i. e. *participate in a piano‐carrying* or *participate in a performance of the Kreutzer sonata*. We are doubtful that this is possible for *perform Hamlet* in English and assure the reader that it most certainly is not for the German translation of this predicate (*Hamlet aufführen*).However, the sentence (18) isn't clearly false, either, and its negation (19) is equally unnatural to describe the situation.

(19) The boys aren't performing *Hamlet*.

This is because, since the plurality of all boys overlaps with (in this case, is a part of) a plurality of which the predicate is true (namely the plurality of all students), the sentence is forced to be undefined by upward homogeneity.

Scenario 3 *Some of the boys together with some of the girls are engaged in the performance.*

Certainly (18), as it stands, isn't true in this scenario, and so it is forced to be undefined by sideways homogeneity, because the predicate is true of a plurality that overlaps with the boys.

Križ (2015a) argues that homogeneity ought to be taken as a fundamental property of lexical predicates in natural language. For reasons of space, we cannot delve into how this is generalised to *n*‐ary predicates, though in light of the above discussion, it bears mentioning that things play out in such a way that a derived unary predicate like *perform Hamlet*, which is formed by combining a binary relation (*perform*) with an atomic individual as an argument in one position (the play *Hamlet*), behaves like a lexical predicate. For a recent revival of the contrary view that it is a logical operator (though not the silent distributivity operator, so that collective predicates can be accounted for as well) that introduces homogeneity, see Bar‐Lev, 2018.

Križ notes two systematic exceptions to the generalisation that homogeneity is a lexical predicate, one in either direction. First, there is a small class of collective predicates that form a principled exception to the rule that lexical predicates show homogeneity: these are predicates that involve measuring a plurality in some way, such as *heavy* and *light* on their collective readings, and *numerous* and *few in number*. Obviously, *numerous* can be true of a large plurality even when it is plainly false of various small subpluralities of that plurality.

Second, there is a class of non‐lexical predicates that systematically shows homogeneity as well, namely phrasally distributive predicates that are derived with the silent distributivity operator *D*. A sentence like (20) has two readings. On its default reading, there is only one pen and five sheets of paper. On the distributive reading, however, there is one pen *per student*. This so‐called *phrasal distributivity* is generally assumed to be introduced by a silent operator *D* (cf. e.g., Champollion, 2010).

(20) The students received a pen.

The phrasally distributive reading of (20) shows homogeneity:

(21) The students *D* received a pen.
**true** if each student received a pen
**false** if no student received a pen
**undef.** otherwise

The sentence is undefined if only some students received a pen, showing homogeneity of the distributive non‐lexical predicate *D received a pen*. This necessitates postulating a meaning for *D* as a function from predicates to predicates that ensures that the output predicate is homogeneous. This sets the silent distributivity operator apart from overt quantifiers (Section 4), and in fact, it may not be appropriate to think of it as a quantifier at all. It appears that the correct notion is rather that the *D* operator takes a predicate *P* and returns a new predicate that has a truth value of a plurality *in virtue of* having that truth value of all of the parts of that plurality:

(22) *D*(*P*)(*a*)
**true** if *P* is true of all parts of *a*
**false** if *P* is false of all parts of *a*
**undef.** otherwise

## 4 HOMOGENEITY AND QUANTIFICATION

This section gives a brief overview of homogeneity effects in quantified sentences. For more detailed descriptions as well as systematic theoretical accounts, the reader is referred to Križ, 2015a, Križ and Spector, 2017, Križ, 2017, and Bar‐Lev, 2018.

### 4.1 Homogeneity removal

In contrast to what we have just seen with the silent distributivity operator, *every*, as a true universal quantifier over atomic individuals, makes homogeneity disappear:

(23) Every student received a pen.
**true** if every student received a pen
**false** if not every student received a pen
**undef.** never

It is difficult to see how it could even be otherwise. For each atomic individual, the predicate *received a pen* is either true or false but never undefined. When restricted to the set of atomic individuals, this predicate is bivalent; it is only trivalent on pluralities. The quantifier *every* is thus being applied to a predicate that on its domain, the set of atomic students is bivalent, and the truth conditions of the universal quantification are simply the classical ones.

However, quantification in natural language is not always over atoms. Plural quantifiers are compatible with collective (readings of) predicates, so they must be quantifying over pluralities:

(24) Two boys carried the piano upstairs.
  "There is a plurality of boys with two members such that that plurality (collectively) carried the piano upstairs."

Löbner (2000) observes that even plural quantifiers, whether in the noun phrase or adverbial, systematically make homogeneity effects disappear: (25) is always either true or false and never undefined.

(25) Two books were published.
  "There is a duality (plurality with two members) of two books such that that duality was published."
**true** if two or more books were published 7We are considering the non‐upper‐bounded reading of the numeral here for simplicity. The upper‐bounded reading is only true if the number of books published by Benfleet is exactly two, and false otherwise.
**false** if at most one book was published
**undef.** never

Note that if only one book was published, then the predicate *was/were published* is not false of all dualities of books. It is not true of any such duality, either, but there are plenty of which it is undefined: just take the one book that was published together with any other book that was not. One might think that the existence of a potential witness of which the predicate is undefined could render the existential quantification undefined, but this is not so in this case; it is still observably plainly false, and (25) is not a gappy sentence.

There seems to be some speaker variation with respect to whether *all* is acceptable with collective action predicates. 8Dowty (1987) does accept this construction, as does Brisson (1998). We ourselves are inclined to agree. Dowty himself already notes that other speakers have rejected such sentences on a collective reading, and this judgment is shared by Champollion (2010: 191f.) and Winter (2001).When acceptable, the addition of *all* interestingly has the effect of removing homogeneity in a limited fashion; it makes the sentence false as soon as there is one part of the plurality of which the predicate is false. To see this, refer again to the scenarios from Section 3 . The addition of *all* causes (26) to be false in Scenario 1 above, because there is a group of boys which is not taking part in any performance of *Hamlet* and of which the predicate is consequently false. The same is true of Scenario 3. 9In the latter case, there is an additional complication. While the negation of (26), (ia), is clearly true in Scenario 1, it seems slightly strange in Scenario 3. We believe that the reason for this is that it tends to trigger the implicature (ib), which is certainly not true, and likely undefined, in Scenario 3.(i)a. Not all the boys are performing *Hamlet*. b. Some of the boys are performing *Hamlet*.However, (26) is still neither true nor false in Scenario 2, because all the boys are participating in the performance, and so the predicate isn't false of any of them; it's undefined of all of them.

(26) All the boys are performing *Hamlet*.

### 4.2 Homogeneity projection

Quantifiers remove homogeneity only, as it were, with respect to the particular argument position that they are filling. With a quantifier in subject position, a definite plural in object position still gives rise to gappiness:

(27) Two publishers accepted the books.
**true** if at least two publishers accepted all the books
**false** if at most one publisher accepted any of the books
**undef.** otherwise, e.g., if one publisher accepted all of the books and a second accepted only some of them

The pattern according to which homogeneity projects, if one so will, through quantifiers was studied experimentally in Križ and Chemla, 2015. They investigated sentences of the form in (28), with a definite plural containing a variable bound by the quantifier, ensuring that it would take low scope. The quantifiers they investigated were *every/all*, *no*, and *exactly two*.

(28) [Quantifier] of the boys found their presents.

To diagnose a gap in a truth‐value judgement task, Križ and Chemla (2015) presented subjects with three answer options: *completely true*, *completely false*, and *neither*. A significant proportion of *neither* responses was diagnostic of undefinedness. The validity of this diagnostic was confirmed by the contrast with control sentences that had *all their presents* instead of the plain definite plural, and which therefore had no gappiness associated with them. For such sentences, subjects did not make use of the *neither* answer. This shows that the *neither*‐responses really reflected a gap rather than some other intuitive notion of closeness to truth. 10What we mean here is the sense in which *all the students are asleep* is closer to being true when most of the students are asleep than it is when only a few students are asleep, despite being false in both cases.


An intuitive formulation of the pattern that emerged is this: the sentence is defined only if it doesn't matter in which direction the undefinedness caused by the definite plural is resolved. If the direction of resolution makes a difference, then the quantificational sentence is undefined. 11This is, of course, closely related to the intuitions behind Strong Kleene logic and supervaluationism.


Consider for illustration the question of how the sentence (29) is evaluated in the two scenarios in (30; assuming that there are three boys).

(29) Every boy found his presents.
(30) Scenario 1: One boy found all of his presents, the second found half, and the third found none.
  Scenario 2: One boy found all of his presents and the other two each found half.

We can schematically represent the extension of the predicate *found his present* in these scenarios as in (31), where we notate the third truth value as ⋆ (1 being truth and 0 being falsity. 12For ease of exposition, this section is written from the perspective of trivalent logic, where there is simply a third truth value besides truth and falsity that a sentence has when it is neither true nor false. A predicate denotation can then be viewed as a function that maps an individual to one of these three truth values. We use a notation of the form *f*=[*x*↦*y*] to indicate that *f* is a function that maps *x* to *y*.

(31) Scenario 1: ⟦found his presents⟧ = $\left[\begin{array}{c} b_{1}\mapsto 1\\ b_{2}\mapsto ⋆\\ b_{3}\mapsto 0 \end{array}\right]$
  Scenario 2: ⟦found his presents⟧ = $\left[\begin{array}{l} b_{1}\mapsto 1\\ b_{2}\mapsto ⋆\\ b_{3}\mapsto ⋆ \end{array}\right]$

What we mean by “resolving undefinedness” is replacing ⋆ with either 1 or 0. So in Scenario 1, we get two possible resolutions:

(32) Resolutions in Scenario 1
  a. $\left[\begin{array}{l} b_{1}\mapsto 1\\ b_{2}\mapsto 1\\ b_{3}\mapsto 0 \end{array}\right]$
  b. $\left[\begin{array}{l} b_{1}\mapsto 1\\ b_{2}\mapsto 0\\ b_{3}\mapsto 0 \end{array}\right]$

If we apply the quantifier *every boy* to these resolutions, it yields the same truth value both times: it is false. This is what we mean when we say that it doesn't matter which way the undefined cases are resolved. If the quantifier yields the same truth value for all resolutions, then that is the truth value of the sentence overall, so it is plainly false in Scenario 1.

In contrast to this, we have the following two resolutions in Scenario 2: 13Note that it is important that all instances of ⋆ get resolved to the same value: we cannot resolve one instance to 0 and the other to 1. (i) Not Resolutions in Scenario 2 a. $\left[\begin{array}{l} b_{1}\mapsto 1\\ b_{2}\mapsto 1\\ b_{3}\mapsto 0 \end{array}\right]$ b. $\left[\begin{array}{l} b_{1}\mapsto 1\\ b_{2}\mapsto 0\\ b_{3}\mapsto 1 \end{array}\right]$

(33) Resolutions in Scenario 2
  a. $\left[\begin{array}{l} b_{1}\mapsto 1\\ b_{2}\mapsto 1\\ b_{3}\mapsto 1 \end{array}\right]$
  b. $\left[\begin{array}{l} b_{1}\mapsto 1\\ b_{2}\mapsto 0\\ b_{3}\mapsto 0 \end{array}\right]$

As for these two resolutions, applying the quantifier *every boy* to them yields different truth values: the quantifier is true of a (33a) but false of (33b). Because it yields different truth value for different resolutions, the quantification is overall undefined in Scenario 2.

Overall, Križ and Chemla, 2015 found the following truth and falsity conditions for (34), which are captured by the principle just discussed.

(34) Every boy found his presents.
**true** if every boy found all of his presents
**false** if at least one boy found none of his presents
**undef.** otherwise

An alternative intuitive method of obtaining the same result is to look at the sentences that are obtained when the definite plural is replaced by an existential and a universal, respectively. If both are true, the sentence with the plain definite is true, and if both are false, it is false; otherwise, undefinedness is obtained.

(35) a. Every boy found some of his presents.
  b. Every boy bound all of his presents.

This method is slightly less general because it is only applicable to sentences with definite plurals in the scope of quantifiers. However, as we will see in Section 5, there are plenty of cases where a predicate is undefined of not only pluralities but also atomic individuals, and this undefinedness needs to project through a quantifier. Some of these cases do not involve a definite plural at all. As such, the replacement method can only be regarded as a convenient heuristic for this particular case.

## 5 VARIETIES OF PARTHOOD

One of Löbner, 2000's (2000) fundamental insights was that homogeneity phenomena are not tied to pluralities per se but arise whenever a property is ascribed to a mereologically complex object. We can observe homogeneity not only with respect to the parthood relation that connects pluralities with their parts but also with respect to material parthood and other, more abstract kinds of parthood.

Consider, for example, a wall, half of which has been painted red while the other half is still untreated. In that case, both (36a) and its negation (36b) are undefined in the typical way of a homogeneity violation.

(36) a. The wall is painted red.
  b. The wall isn't painted red.

For an entirely abstract example, consider a book in its capacity as a structured linguistic entity (a sequence of sentences, if you will). Faced with a book that contains some brilliant and some stupid chapters, we would not say that either (37a) or (37b) are true.

(37) a. The book is intelligently written.
  b. The book isn't intelligently written.

Homogeneity with respect to two kinds of parthood can co‐occur in one sentence.

(38) The books are intelligently written.
**true** if all books are intelligently written throughout
**false** if no book contains notable intelligently written parts
**undef.** otherwise

If some books are intelligently written and others are not, then (38) is undefined due to homogeneity with respect to the parthood relation that holds between pluralities and the individuals they are made up of (following Link (1983), this is often called *individual parthood*). If, on the other hand, all the books contain some intelligently written passages and some very dumb passages, then it is homogeneity with respect to the abstract parts of books that is responsible for undefinedness.

Quantifiers over individuals—which is what linguists generally talk about when they speak of quantifiers without further qualification—remove homogeneity only with respect to individual parthood. What happens if the predicate is also homogeneous with respect to another parthood relation is quite analogous to the case where we have a definite plural in object position: now we have a predicate that is sometimes undefined even of atoms, and not only of pluralities, and this undefinedness “projects” according to the pattern discussed in Section 4.2.

(39) All the books are intelligently written.
**true** if all the books are intelligently written throughout
**false** if at least one book doesn't contain intelligent passages
**undef.** otherwise

But there are other quantifiers, both adnominal and adverbial, that remove homogeneity with respect to different kinds of parthood. For a simple illustration, let us have a look at various quasi‐universal quantifiers, which work analogously to adnominal and adverbial *all*. In English, adnominal *whole* is essentially a general‐purpose homogeneity remover (except for individual parthood). 14Why *whole* is an adjective that seemingly forms a constituent with the noun below the definite article is quite mysterious. The same happens with *most* in German (*die meisten*), which is usually simply analysed as a complex determiner. See also Moltmann, 1997 on *whole*.

(40) a. The whole wall is(n't) painted red.
  b. The whole book is(n't) intelligently written.

In the adverbial domain, we find more variation. For the spacially flavoured case of (36), *everywhere* serves as an adverbial homogeneity remover.

(41) a. The wall is(n't) painted everywhere.
  b. The forest is(n't) dense everywhere.

For the abstract (37), one may regard *throughout* as an adverbial homogeneity remover. It is unclear to us why it sounds slightly odd under negation.

(42) a. The book is written intelligent throughout.
  b. The book isn't written intelligently throughout.

In German, there is an adverbial homogeneity remover *durchgehend*, which is appropriate for this case and perfectly natural under negation.

(43) a. Das Buch ist (nicht) durchgehend intelligent geschrieben.
*the book is (not) throughout intelligently written*

This cues us into another relevant observation: there is considerable cross‐linguistic variation in the inventory of quantificational homogeneity removers. For example, while English makes a distinction between *all*, which is reserved for individual parthood, and *whole*, other languages employ the same lexical item in both cases.

When homogeneity with respect to different parthood relations co‐occurs, it can be removed separately. We also saw such a case in (39) above, where *all* removed homogeneity with respect to the plurality of books but not with respect to the parts of the individual books. In a sense, we can speak of there being two layers of homogeneity: an outer layer, which concerns the relation between the plurality and its constituent atoms and an inner layer, which concerns the abstract parts that these atoms (which are atoms with respect to individual parthood but not with respect to this abstract parthood relation) have in turn. The inner layer can be removed on its own or in addition to the outer layer:

(44) The books are intelligently written throughout.
**true** if all the books are intelligently written throughout
**false** if all the books contain at least some stupid parts
**undef.** otherwise
(45) All the books are intelligently written throughout.
**true** if all the books are intelligently written throughout
**false** if at least one book contains at least some stupid parts
**undef.** never

It may be worth noting that an adnominal modifier can only be used to remove the outermost layer of homogeneity: *whole* refuses to occur in a plural noun phrase.

(46) a. #The whole books are intelligently written.
  b. #All the whole books are intelligently written.

## 6 NO ROLE FOR THE NOUN PHRASE

It has occasionally been suggested that homogeneity effects are rooted in the meaning of the definite plural noun phrase (Breheny, 2005; Büring & Križ, 2013; Magri, 2014), rather than the predicate. While particular instantiations of this view may face additional objections, the following arguments pose problems for this whole family of analyses.

We noted in Section 3 that some predicates, namely those that involve some kind of measuring of a plurality such as *heavy* and *numerous*, are exempt from the homogeneity constraint. It is not clear why this is so, apart from the functional point that if they were constrained by homogeneity, their meanings would appear rather odd and not very useful. However, on the view that homogeneity comes from the definite plural, the existence of nonhomogeneous sentences like (47a) is entirely inexplicable.

(47) The students are numerous.

Once we look, with Löbner, at homogeneity beyond individual parthood, we also find that no definite plural is necessary at all to trigger it. First, homogeneity with respect to abstract parthood appears with proper names:

(48) *Decline and Fall* is intelligently written.
**true** if the whole of *Decline and Fall* is intelligently written
**false** if no part of it is
**neither** otherwise

Second, we find homogeneity with respect to these other relations of parthood remaining as an inner layer of homogeneity after removing the outer layer with a quantifier, as in (39) above, and indeed also under the singular universal quantifier *every*: (49) has the same truth and falsity conditions as (39).

(49) Every book is intelligently written.

Clearly the appearance of homogeneity has to do with the fact that we are ascribing a particular predicate to a particular sort of object. As far as we can discern, a view on which homogeneity is somehow rooted in the noun phrase has no way to make sense of this and also cannot deal with the separate removal of different layers of homogeneity by adverbials (*all* and *throughout*, such as in (50).

(50) The books are all intelligently written throughout.

## 7 HOMOGENEITY AND EXCEPTION TOLERANCE

The above picture, where plural predication has strictly universal truth and falsity conditions, is actually a simplification of what we observe in actual communication. It has long been noticed that depending on the context and purposes of the conversation, plural predication can quite readily admit of exceptions. Following Brisson, 1998, this phenomenon is most frequently referred to as *non‐maximality*. 15See also Lasersohn, 1999; Malamud, 2012; Križ, 2016; Križ and Spector, 2017.


Križ (2016) presents the following example, where the context is such that the exception is plausibly considered irrelevant:

(51) Context: *All the professors in the jury are smiling in response to a job talk, except for Prof. Smith, who is known to always have a dour expression. He never smiles, so this doesn't mean much.*
  The professors are smiling.

He argues that non‐maximality is intimately linked to homogeneity: it is shown by all homogeneous constructions, and it disappears when homogeneity is removed by a quantifier. This can be seen in the dual effect of *all*: it is both a homogeneity remover and what has been called a *slack regulator*, which is to say it removes the potential for non‐maximality. The sentence in (52), with *all*, is not only nonhomogeneous but also does not allow for an exception: it is just clearly and plainly false in the situation at hand.

(52) Context: *All the professors in the jury are smiling in response to a job talk, except for Prof. Smith, who is known to always have a dour expression. He never smiles, so this doesn't mean much.*
  #All the professors are smiling.

This link is in need of a principled explanation, and two types of theories have been proposed. According to the first (Križ, 2016), homogeneity is semantic, and non‐maximality is post‐semantic and explanatorily downstream of homogeneity. The second approach (Bar‐Lev, 2018; Križ & Spector, 2017) has it that both homogeneity and non‐maximality are consequences of the same underlying mechanism that involves, in one way or another, the manipulation of a set of meaning alternatives of some sort (i.e., neither is downstream of the other).

On both approaches, it is a conceptual necessity that whatever causes homogeneity to disappear also removes non‐maximality, thus explaining the correlation and the effect of *all*. However, a further exploration of this topic is, unfortunately, beyond the scope of this article.

## 8 BEYOND THE INDIVIDUAL DOMAIN

Homogeneity effects, or something looking strikingly alike, can be observed in a number of additional domains. 16This section serves only to give a brief overview—for reasons of space, we have to refer the reader to the literature for arguments in support of the descriptions made here.


It can be shown that for all intents and purposes, the conditional *if* *ϕ*, *then not* *ψ* functions as the negation of *if* *ϕ*, *then* *ψ* (e.g., Higginbotham, 2003; Križ, 2015a). It is then easy to see that the truth conditions of a conditional and its negation are not complementary. (53) conveys that in all possible situations where Nina comes to the party, barring perhaps very exceptional and unusual events, Adam is happy.

(53) If Nina comes to the party, Adam will be happy.

Its negation (54), correspondingly, means that in all possible situations where Nina comes to the party, Adam is not happy.

(54) If Nina comes to the party, Adam won't be happy.

But what if Nina's coming has no influence on Adam's happiness, and so in some possible situations where she comes, he is happy, and in others, he is not, dependent on other circumstances? Then neither of these two conditionals is appropriate, and a *well*‐answer is appropriate, just as for homogeneity:

(53) Context: *Nina's coming to the party may make Adam happy, but is not guaranteed to (depending on other factors).*
  A: If Nina comes to the party, Adam will be happy.
  B: Well…He might be.

This phenomenon has received quite some attention under the slightly different framing of *conditional excluded middle* (e.g., Stalnaker, 1981; von Fintel, 1999; Gajewski, 2005; Klinedinst, 2010). However, it is attractive to understand it in terms of homogeneity. First of all, this fits well with the idea that conditionals are, on some level, plural predication over pluralities of possible worlds (e.g., Schlenker, 2004; Klinedinst, 2007): *if ϕ, then ψ* is taken to mean that *ψ* *is the case in **the** accessible* *ϕ*‐*worlds*. Second, it provides a new way to make sense of the well‐known exception tolerance of conditionals (Gillies, 2007; Klecha, 2015; Lewis, 1973; Moss, 2012; von Fintel, 2001; Willer, 2017): Križ (2014) argues that this is simply the non‐maximality that accompanies homogeneity.

Sentences with embedded questions also have negations with noncomplementary truth conditions:

(56) a. Agatha knows who was at the party.
  ⇝ Agatha is fully informed.
  b. Agatha doesn't know who was at the party.
  ⇝ She has pretty much no idea.

It was first pointed out by Gajewski (2005) that this looks rather like homogeneity, which prompted Križ (2015b) and Cremers (2015) to develop explicit theories analysing it as such.

Bare plural generics also show homogeneity effects like sentences with definite plurals (Cohen, 2004; Löbner, 2000; Magri, 2012) and are furthermore famously exception tolerant. A fully explicit analysis of homogeneity in generics that fits with the picture of homogeneity rooted in the ascription of a predicate to an object that has parts is, however, still missing; only a programmatic sketch is suggested in Križ 2015a: §7.

(57) a. Dogs are intelligent.
  ⇝ Pretty much all dogs are intelligent.
  b. Dogs aren't intelligent.
  ⇝ Pretty much no dogs are intelligent.

## 9 CONCLUSION AND OUTLOOK

In this paper, we have attempted to give the reader a brief introduction to the phenomenon of homogeneity effects in natural language, as a particular way in which sentences where a property is ascribed to a plurality of objects are sometimes neither true nor false. The conditions under which this is the case have been reasonably well‐identified and interact with quantification according to a particular established pattern.

We then continued with a presentation of homogeneity phenomena beyond the core case of plural predication: first with respect to other parthood relations, and then beyond the nominal domain entirely, where the recognition of homogeneity as a pervasive aspect of natural language semantics may ultimately provide a perspective for a unified analysis across various constructions that were hitherto largely analysed in quite different terms.

Ongoing—and needed future—work in this area falls broadly into two categories. The first contains a further precisification of the formal and conceptual groundwork regarding the core phenomenon centred around plural noun phrases, wrestling, not least, with the question of why such an apparently *sui generis* gappiness phenomenon should exist and why it should be coupled with exception tolerance (as mentioned above in Section 7). Recent work by Križ and Spector, 2017 and Bar‐Lev, 2018 falls into this category.

The second strand of inquiry concerns the extension of this treatment to phenomena beyond definite plurals. While empirical parallels have been noted (as per Section 8), a cogent formal treatment of these cases that provides an appropriate conceptual unification with the core phenomenon around plurals has, in many cases, not been fully worked out or is lacking entirely. Additionally, there is still an open question of whether there are yet further phenomena that could be brought under the same umbrella—most recently, free‐choice permission has been named as a candidate (Bar‐Lev, 2018; Goldstein, 2019).

## References

[lnc312350-bib-0001] Bar‐Lev, M. (2018). Free choice, homogeneity and innocent inclusion. (PhD thesis), Hebrew University of Jerusalem.

[lnc312350-bib-0002] Breheny, R. (2005). Exhaustivity, homogeneity, and definiteness. In *Proceedings of the fifth Amsterdam colloquium* (Dekker P., & Franke M., Eds.). Amsterdam: ILLC, pp. 59–65.

[lnc312350-bib-0003] Brisson, C. (1998). Distributivity, maximality, and floating quantifiers. (PhD thesis), Rutgers.

[lnc312350-bib-0004] Büring, D. , & Križ, M. (2013). It's that and that's it! Exhaustivity and homogeneity presuppositions in clefts (and definites). Semantics and Pragmatics, 6, 6–1.

[lnc312350-bib-0005] Champollion, L. (2010). Parts of a ahole: Distributivity as a bridge between aspect and measurement. (PhD thesis), University of Pennsylvania.

[lnc312350-bib-0006] Cohen, A. (2004). Generics and mental representations. Linguistics and Philosophy, 27, 529–556.

[lnc312350-bib-0007] Cremers, A. (2015). Homogeneity and quantificational variability with embedded questions. Ms. LSCP.

[lnc312350-bib-0008] Cremers, A. , Križ, M. , & Chemla, E. (2017). Probability judgments of gappy sentences, Linguistic and psycholinguistic approaches to implicatures and presuppositions. Cham:Palgrave, pp. 111–150.

[lnc312350-bib-0009] Dowty, D. (1987). A note on collective predicates, distributive predicates and all. Proceedings of ESCOL, 86, 97–115.

[lnc312350-bib-0010] Fodor, J. D. (1970). The linguistic description of opaque contexts. (PhD Thesis), MIT, Cambridge, Mass.

[lnc312350-bib-0011] Gajewski, J. (2005). Neg‐Raising: Polarity and resupposition. (PhD thesis), MIT.

[lnc312350-bib-0012] Gillies, A. (2007). Counterfactual scorekeeping. Linguistics and Philosophy, 30, 329–360.

[lnc312350-bib-0013] Goldstein, S. (2019). Free choice and homogeneity paper presented at meaning in action. Oxford:All Souls College.

[lnc312350-bib-0014] Higginbotham, J. (2003). Conditionals and compositionality. Philosophical Perspectives, 17(1), 181–194.

[lnc312350-bib-0015] Klecha, P. (2015). Two kinds of sobel sequences: Precision in conditionals. In *Proceedings of [WCCFL 32]* (Steindl U., Borer T., Fang H., García Pardi A., Guekguezian P., Hsu B., O'hara C., & Ouyang I. C., Eds.). Somerville, MA: Cascadilla Press Cascadilla Proceedings Project, pp. 131–140.

[lnc312350-bib-0016] Klinedinst, N. (2007). Plurality and possibility. (PhD thesis), UCLA.

[lnc312350-bib-0017] Klinedinst, N. (2010). Quantified conditionals and conditional excluded middle. Journal of Semantics, 28(1), 149–170.

[lnc312350-bib-0018] Križ, M. (2014). Conditionals, monotonicity, and definite descriptions. Poster presented at semantics and philosophy in Europe 7. Berlin, Germany.

[lnc312350-bib-0019] Križ, M. (2015a). Aspects of homogeneity in the semantics of natural language. (PhD thesis). Amsterdam: University of Vienna.

[lnc312350-bib-0020] Križ, M. (2015b). Homogeneity, trivalence, and embedded questions. In *Proceedings of the 20th Amsterdam colloquium* (Brochhagen T., Roelofsen F., & Theiler N., Eds.)

[lnc312350-bib-0021] Križ, M. (2016). Homogeneity, non‐maximality, and ‘all’. Journal of Semantics, 33(3), 493–539.

[lnc312350-bib-0022] Križ, M. (2017). Bare plurals, multiplicity, and homogeneity. Ms. IJN.

[lnc312350-bib-0023] Križ, M. , & Chemla, E. (2015). Two methods to find truth‐value gaps and their application to the projection problem of homogeneity, Vol. 23, pp. 205–248.

[lnc312350-bib-0024] Križ, M. , & Spector, B (2017). Interpreting plural predication. Homogeneity and non‐maximality. Ms. IJN. 10.1007/s10988-020-09311-w PMC8550301 34720264

[lnc312350-bib-0025] Krifka, M. (1996). Pragmatic strengthening in donkey sentences and plural predications. In *Proceedings of SALT VI* (Galloway T., & Spence J., Eds.). Ithaca, NY: Cornell University, pp. 136–153.

[lnc312350-bib-0026] Lasersohn, P. (1999). Pragmatic halos. Language, 3(75), 522–551.

[lnc312350-bib-0027] Lewis, D. (1973). Counterfactuals. Oxford:Blackwell.

[lnc312350-bib-0028] Link, G. (1983). The logical analysis of plurals and mass terms: A lattice‐theoretical approach, Meaning, use and interpretation of language. Berlin:de Gruyter.

[lnc312350-bib-0029] Löbner, S. (2000). Polarity in natural language: Predication, quantification and negation in particular and characterizing sentences. Linguistics and Philosophy, 23, 213–308.

[lnc312350-bib-0030] Magri, G. (2012). No need for a dedicated theory of the distribution of readings of English bare plurals, Proceedings of SALT 22. Ithaca, NY: CLC, pp. 383–402.

[lnc312350-bib-0031] Magri, G. (2014). An account for the homogeneity effects triggered by plural definites and conjunction based on double strengthening, Pragmatics, semantics and the case of scalar implicatures, palgrave studies in pragmatics, language and cognition. Houndsmills:Palgrave Macmillan, pp. 99–145.

[lnc312350-bib-0032] Malamud, S. (2012). The meaning of plural definites: A decision‐theoretic approach. Semantics and Pragmatics,5, 1–58.

[lnc312350-bib-0033] Moltmann, F. (1997). Parts and wholes in semantics Oxford University Press. Oxford:New York.

[lnc312350-bib-0034] Moss, S. (2012). On the pragmatics of counterfactuals. Noûs, 46(3), 561–86.

[lnc312350-bib-0035] Schlenker, P. (2004). Conditionals as definite descriptions (a referential analysis). Research on Language and Computation, 2(3), 417–462.

[lnc312350-bib-0036] Schwarz, F. (2013). Maximality and definite plurals—Experimental evidence. In *Proceedings of [Sinn und Bedeutung 17]* (Chemla E., Homer V., & Winterstein G., Eds.). Paris, pp. 509–526.

[lnc312350-bib-0037] Schwarzschild, R. (1996). Pluralities. Dordrecht; Boston:Kluwer Academic.

[lnc312350-bib-0038] Spector, B. (2013). Homogeneity and plurals: From the strongest meaning hypothesis to supervaluations. Presented at Sinn und Bedeutung. 18.

[lnc312350-bib-0039] Stalnaker, R. (1981). A defense of conditional excluded middle, Ifs: Conditionals, belief, decision, chance and time. Dordrecht:Reidel, pp. 87–104.

[lnc312350-bib-0040] von Fintel, K. (1999). NPI licensing, strawson entailment, and context dependency. Journal of Semantics, 16(2), 97–148.

[lnc312350-bib-0041] von Fintel, K. (2001). Counterfactuals in a dynamic context, Ken Hale: A life in language. Cambridge:MIT Press, pp. 123–152.

[lnc312350-bib-0042] Willer, M. (2017). Lessons from sobel sequences. Semantics and Pragmatics, 10.

[lnc312350-bib-0043] Winter, Y. (2001). Flexibility principles in Boolean semantics. Cambridge, MA:MIT Press.

[lnc312350-bib-0044] Zehr, J (2014). Vagueness, presupposition and truth‐value judgments. (PhD thesis), École Normale Supérieure de Paris.

